# Coupled effects of nanoparticle surface roughness and grafting architecture on mechanical reinforcement in polymer nanocomposites

**DOI:** 10.1088/1361-6528/ae9d4b

**Published:** 2026-09-02

**Authors:** Haoyu Wang, Zhangke Yang, Zhaoxu Meng

**Affiliations:** Department of Mechanical Engineering, Clemson University, Clemson, SC 29634, United States of America

**Keywords:** polymer nanocomposites, nanoparticle surface roughness, grafting architecture, mechanical reinforcement, coarse-grained molecular dynamics, topological constraints

## Abstract

Polymer nanocomposites derive their mechanical performance from nanoscale interfacial mechanisms that govern stress transfer, nanoconfinement, and deformation resistance. Among these mechanisms, nanoparticle surface geometry, interfacial interactions, and grafting architecture are particularly important, yet their coupled effects on reinforcement remain insufficiently understood. Here, coarse-grained molecular dynamics simulations are used to investigate polymer nanocomposites containing a rigid spherical nanoparticle with either smooth or sinusoidally rough surface geometry. Non-grafted and grafted systems with different grafting architectures are compared under weak and strong interfacial interactions. Under weak interfacial interactions, changes in surface roughness and grafting produce only modest differences in the tensile response. In contrast, under strong interactions, higher-amplitude roughness produces higher elastic moduli than the smooth case, consistent with more restricted interfacial polymer mobility and more effective load transfer. Grafting further amplifies reinforcement by creating permanent nanoparticle–polymer connectivity and increasing the mean number of topological constraints. Primitive-path analysis shows that grafting produces a larger topological shift than surface roughness. Beyond uniform grafting, the spatial organization of grafting sites provides additional tunability. Clustered grafting induces directional mechanical response, while targeted placement of grafting sites at surface apexes of surface-roughened nanoparticle more effectively couples tethered polymer chains to surface roughness features. These findings demonstrate that nanocomposite reinforcement arises from a cooperative interplay among surface roughness, interfacial interaction, and grafting architecture. The results provide molecular-level design principles for engineering polymer nanocomposites with improved interfacial load transfer, stiffness, and tailored mechanical performance.

## 1. Introduction

Polymer nanocomposites provide an effective route for improving stiffness, strength, damping, and functionality through the incorporation of nanoscale fillers [1–3]. Their macroscopic mechanical response depends not only on filler loading, size, and dispersion, but also critically on the organization and dynamics of polymer chains within the polymer–filler interphase. In this region, chain packing, segmental mobility, adsorption, and stress transfer differ from those in the bulk matrix, leading to distinct mechanical behavior [4–12]. Both experimental and simulation studies have shown that polymer layers adjacent to nanoparticle surfaces exhibit altered mobility and contribute significantly to overall reinforcement, underscoring the importance of interfacial design in the mechanics of polymer nanocomposites [6–12].

Among the interfacial characteristics, nanoparticle surface topography plays a key role by modifying local contact geometry, enhancing polymer confinement, and potentially hindering interfacial chain sliding during deformation [13–16]. Recent studies also indicate that the mechanical effect of nanoparticle surface roughness depends strongly on the surrounding interfacial environment, particularly the strength of polymer–nanoparticle interactions [14–16]. For example, in polymethyl methacrylate (PMMA) nanocomposites, variations in surface geometry influence stiffness, damping, and deformation response, while stronger interfacial interactions generally enhance reinforcement [14–16]. More broadly, studies of attractive polymer–nanoparticle systems have shown that interfacial strength, adsorption, and confinement govern the efficiency of stress transfer from matrix to filler [9–12, 17]. These findings suggest that the mechanical role of surface roughness must be evaluated in conjunction with interfacial coupling rather than as an isolated parameter.

Polymer grafting provides an additional means of tuning interfacial coupling by introducing covalent bonding and modifying the structure of chains near the nanofiller surface [18–21]. Polymer grafting can be prepared either by attaching preformed functionalized chains [19] or by growing polymer chains directly from surface-immobilized initiators using controlled radical polymerization techniques such as atom transfer radical polymerization and reversible addition–fragmentation chain transfer polymerization [20, 21]. Grafted systems exhibit altered chain interpenetration, conformations, rheological behavior, and entanglement characteristics, all of which influence stress transfer across the interface [22–31]. These effects depend on parameters such as grafting density, chain length, nanoparticle size, and matrix molecular weight [22–31]. Additionally, comparisons between covalently bonded and physically adsorbed interfaces have demonstrated the importance of polymer grafting in governing interfacial dynamics and relaxation behavior [17].

Despite these advances, the combined influence of surface roughness and grafting architecture remains insufficiently understood within a unified polymer nanocomposite system. Most prior studies have focused on either surface topography or grafting independently, making it difficult to determine how roughness-dependent mechanical trends evolve when permanent interfacial connectivity is introduced. This interplay is inherently complex, as interfacial interaction strength, constrained chain dynamics, and network topology can all contribute to macroscopic mechanical response [23–27].

In this work, we use coarse-grained (CG) molecular dynamics (MD) simulations to systematically investigate PMMA nanocomposites containing a rigid nanoparticle with either a smooth or a sinusoidally rough surface geometry. We examine non-grafted and grafted systems under weak and strong interfacial interactions and further vary the grafting architecture through uniform, clustered, and apex-targeted distributions of grafting sites. This framework enables systematic evaluation of how nanoparticle surface geometry, interfacial interaction strength, and grafting architecture jointly influence interfacial polymer mobility, global topological constraints, and mechanical reinforcement. We note that variations in surface geometry also alter the accessible surface area and effective grafting density. Thus, comparisons among the nanoparticle systems are interpreted as coupled effects rather than as isolated dependences on a single factor.

## 2. Models and methods

### 2.1. Model setup of the nanocomposite

**Figure 1.**
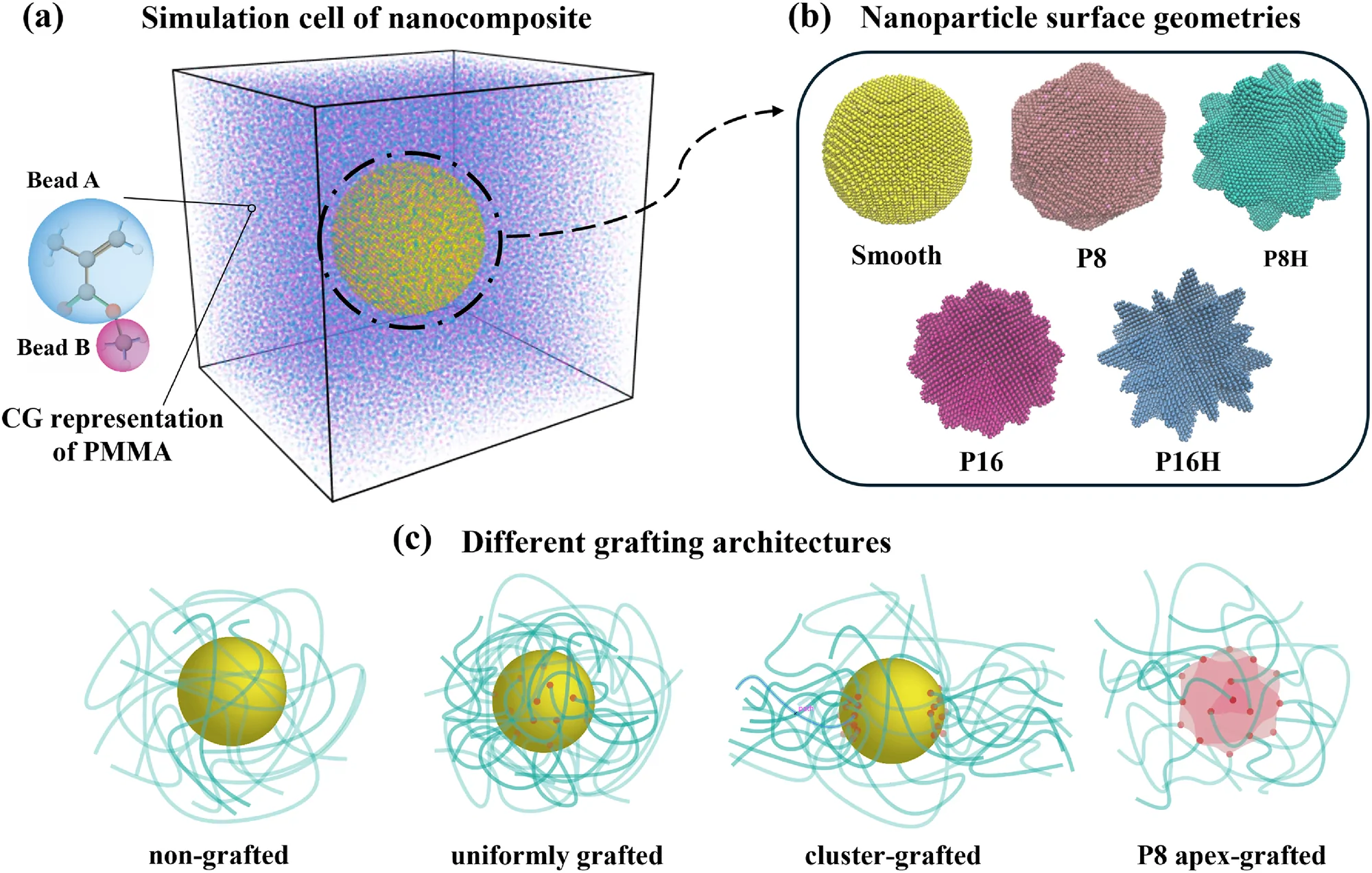
Model setup of the nanoparticle–PMMA nanocomposites and schematic representations of the interfacial architectures. (a) Simulation cell containing a nanoparticle embedded in a PMMA matrix. The dashed circle identifies the nanoparticle-centered interfacial region, and the inset illustrates the two-bead-per-monomer CG representation of PMMA, in which bead A represents the backbone, and bead B represents the side group in a PMMA repeat unit. (b) Nanoparticle surface geometries considered in this study: smooth, P8, P8H, P16, and P16H. (c) Schematic representations of the non-grafted, uniformly grafted, cluster-grafted, and P8 apex-grafted architectures. Polymer chains are represented by continuous lines to illustrate their spatial arrangement and grafting topology. In the grafted systems, the highlighted red beads are nanoparticle surface beads selected as grafting sites.

The nanocomposite system considered in this work consists of a single nanoparticle with systematically varied surface geometry embedded in a PMMA matrix, shown in figure 1(a). The nanoparticle is modeled as a collection of beads arranged on a simple cubic lattice and treated as a rigid body due to its significantly higher stiffness relative to the polymer matrix. The PMMA matrix is represented using a previously developed CG model in which backbone and side-group units are mapped to individual CG beads [32], as shown in figure 1(a).

The equilibrated simulation cell has dimensions of approximately $175 \times 170 \times 170 {\unicode{x00C5}}^3$ with periodic boundary conditions applied in all directions. The nanoparticle has an equivalent diameter of $100{\unicode{x00C5}},$ with its total volume held constant across all surface geometries. Unless otherwise specified, polymer chains are 100 units long, and the simulation cell contains approximately 230 PMMA chains for all the systems considered in this study to maintain a consistent weight fraction of nanoparticle at 0.5.

The relatively high nanoparticle loading is adopted to emphasize polymer behavior within the interphase and to focus on the effects of surface roughness and grafting under controlled conditions. This model is not intended to capture the full complexity of experimentally dispersed nanocomposites, where filler loading, agglomeration, and interparticle spacing can vary substantially, as shown in previous studies of nanocomposites [33–35]. The mechanisms identified here are most relevant to polymer regions adjacent to filler surfaces or within locally confined interparticle domains. The molecular-level insights obtained here may also inform continuum descriptions of nanocomposites, including models or theoretical frameworks [36–38] that explicitly account for interphase behavior.

Surface geometry is introduced in a controlled manner by applying sinusoidal perturbations to an initially smooth surface. Two sinusoidal periodicities and two amplitudes are considered, yielding five surface geometries: Smooth, P8, P16, P8H, and P16H, as shown in figure 1(b). Here, P8 and P16 denote different periodicities, while the suffix H indicates a higher amplitude. The standard and high amplitudes are 5 and 15 Å, respectively. Because these perturbations modify both local curvature and accessible surface area, we consider these different nanoparticles with distinct surface geometries rather than variations of a single roughness parameter. Among the geometries considered, P16H exhibits the most pronounced surface roughness, combining the larger perturbation amplitude with smaller period length.

For each nanoparticle surface geometry, both non-grafted and grafted interfacial configurations are examined (figure 1(c)). In the non-grafted systems, PMMA chains are packed around the nanoparticle without covalent attachment. In the grafted systems, every PMMA chain is covalently tethered once to the nanoparticle surface through a covalent bond between a selected nanoparticle surface bead and bead B of a terminal PMMA repeat unit. Thus, the number of grafted chains equals the total number of polymer chains, and no untethered or free matrix chains are present. Grafting sites are assigned using a geometry-dependent surface selection procedure that enables control over their spatial distribution. In the uniformly grafted systems, accessible nanoparticle surface beads are selected to produce an approximately uniform distribution of grafting sites.

In the cluster-grafted configuration, the grafting sites are concentrated within localized surface domains, creating a spatially heterogeneous interfacial architecture. In the apex-grafted configuration, the grafting sites are selectively placed at the convex apex areas of the P8 surface, as illustrated in figure 1(c). Because only a subset of nanoparticle surface beads is accessible for grafting, a substantial portion of the surface remains available for nonbonded polymer contact and adsorption. Nonbonded interactions between nanoparticle and polymer beads are retained for all non-covalently bonded bead pairs. Since the accessible surface area differs among the nanoparticle architectures, the effective grafting density also varies and is not independently controlled. The present comparisons, therefore, probe the coupled effects of surface roughness and grafting configurations at fixed overall composition rather than isolating grafting density as an independent variable.

The nonbonded interaction is described by a 12–6 Lennard-Jones potential,

\begin{equation*}{E_{{\mathrm{np}}}} = 4{\varepsilon _{{\mathrm{np}}}}\left[ {{{\left( {\frac{{{\sigma _{{\mathrm{np}}}}}}{r}} \right)}^{12}} - {{\left( {\frac{{{\sigma _{{\mathrm{np}}}}}}{r}} \right)}^6}} \right]\end{equation*}

where ${\varepsilon _{{\mathrm{np}}}}$ denotes the nanoparticle–polymer interaction strength and ${\sigma _{{\mathrm{np}}}} = 4.5{{ {\unicode{x00C5}} }}$ is the zero-crossing distance. Two interaction strengths are considered to represent weak and strong interfacial interaction, with ${\varepsilon _{{\mathrm{np}}}} = 0.5{ }$ and 2.5 kcal mol^−1^, respectively. These two values are consistent with our PMMA nanocomposite studies [15, 16]. We note that varying ${\varepsilon _{{\mathrm{np}}}}{ }$ modulates interfacial adhesion as well as local adsorption, packing, and dynamic behavior.

### 2.2. Simulation protocol

All simulations are performed using LAMMPS with a uniform timestep of 4 fs [39]. The initial nanocomposite system is first relaxed using a soft potential to remove unphysical overlaps with a Langevin thermostat and NVE/limit integration. Then, the systems are equilibrated at very high temperature for 0.2 ns to expedite the restoration of the correct interaction potential. Afterward, a multistep NPT protocol is applied, consisting of high-temperature relaxation, pressure-assisted densification, cooling to 300 K with simultaneous pressure release, and a final thermal-cycling step to fully equilibrate the system, consistent with our previous study [15]. This procedure ensures consistent initial states across all surface geometries, grafting architectures, and interfacial interactions.

Following equilibration, uniaxial tensile deformation is applied along the x-direction at a constant engineering strain rate of $5\, \times \,{10^8}{ }{{\text{ s}}^{ - 1}}$. The temperature is maintained at 300 K, while zero pressure is imposed in the transverse directions using the NPT ensemble. The engineering stress–strain response is recorded during deformation, and the elastic modulus is extracted from the initial linear regime (strain between 0 and 0.01), consistent with our prior work [15, 16]. For each system, the elastic modulus is calculated independently from three separately prepared and equilibrated simulations. The reported modulus is the mean of the three independently determined values, and the error bars represent their standard deviation. Because all systems are subjected to an identical deformation protocol, the tensile response is compared to assess how nanoparticle surface roughness, grafting architecture, and interfacial interaction strength influence mechanical behavior within the present model.

Simple shear deformation is imposed by continuously changing the appropriate tilt factor of the triclinic simulation cell at the same nominal engineering strain rate used for uniaxial tension. During deformation, the temperature is maintained at 300 K, while the normal pressure perpendicular to the shear plane is maintained at 0 atm. The shear stress is obtained from the corresponding off-diagonal component of the stress tensor. The same deformation protocol is used for all shear simulations to enable direct comparison among the different loading planes.

Topological analysis is carried out using the Z1+ primitive-path framework [40]. In this method, the primitive-path network is obtained by progressively shortening chain contours while fixing chain ends, preserving chain uncrossability, and yielding a shortest disconnected-path representation of the entanglement network [40]. Primitive-path analysis has been widely used to characterize entanglement structure in polymer systems [41, 42].

In the present study, two quantities reported by Z1+ are used for comparison: the mean primitive-path contour length, ${L_{{\mathrm{pp}}}}$, and the mean number of kinks per chain, Z, defined as

\begin{equation*}{L_{{\mathrm{pp}}}} = \left( {\frac{1}{{{N_c}}}} \right)\sum\limits_{i = 1}^{{N_c}} {{L_{{\mathrm{pp}},i}}} \end{equation*}

\begin{equation*}Z = \left( {\frac{1}{{{N_c}}}} \right)\mathop \sum \limits_{i = 1}^{{N_c}} {Z_i}\end{equation*}

where ${N_c}$ is the total number of polymer chains in the simulation cell, ${L_{{\mathrm{pp}},i{ }}}$ is the primitive-path contour length of each chain i, and ${Z_i}{\text{ }}$ is the corresponding number of kinks.

Within this framework, Z is used as a comparative measure of the mean number of topological constraints per chain, whereas ${L_{{\mathrm{pp}}}}$ describes the geometry and contractibility of the resulting primitive-path network [40]. ${L_{{\mathrm{pp}}}}$ should be interpreted as a complementary structural descriptor rather than as an independent measure of entanglement density. These descriptors are commonly interpreted in the context of tube-model and reptation-based descriptions of entangled polymer dynamics, where chain uncrossability and topological constraints govern stress relaxation and deformation resistance [41].

Unless otherwise stated, these quantities are computed over the full polymer population in the equilibrated simulation cell. Therefore, they are interpreted as global comparative descriptors of the primitive-path network rather than spatially resolved measures of interfacial entanglement. For each system, Z and ${ }{L_{{\mathrm{pp}}}}$ are calculated separately from three independently prepared and equilibrated configurations. The reported values are the means of the three configuration-level results, and the error bars represent their standard deviations.

## 3. Results and discussion

### 3.1. Effects of surface roughness and grafting on mechanical response

**Figure 2.**
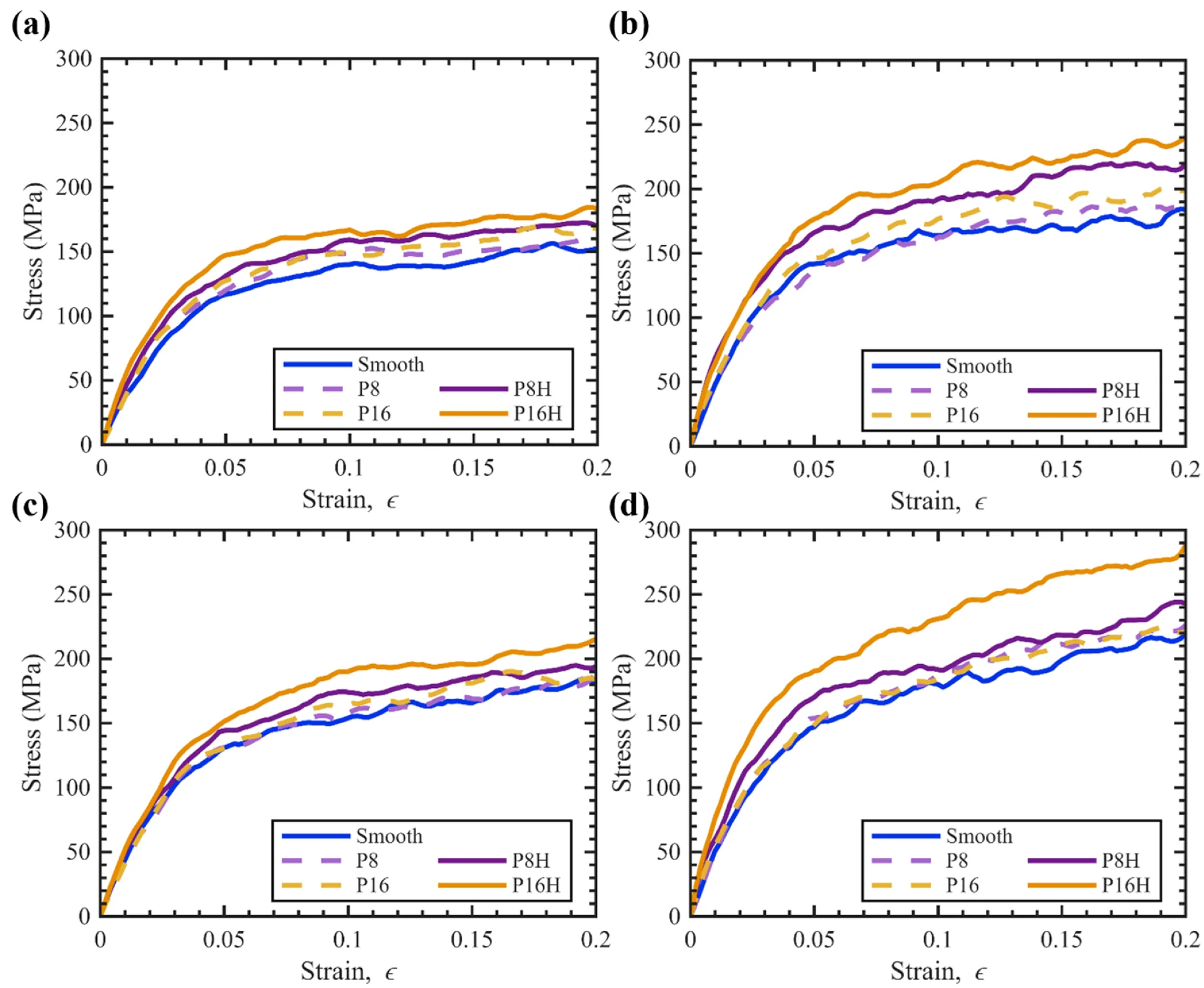
Engineering stress–strain curves of nanoparticle-PMMA nanocomposites with varying surface roughness, grafting configurations, and interfacial interactions. Results are shown for (a) non-grafted systems with weak interaction (${\varepsilon _{\mathrm{np}}}$ = 0.5 kcal/mol), (b) non-grafted systems with strong interaction (${\varepsilon _{\mathrm{np}}}$ = 2.5 kcal/mol), (c) grafted systems with weak interaction, and (d) grafted systems with strong interaction.

Figure 2 compares the engineering stress–strain curves of nanocomposites with different nanoparticle surface geometries under uniaxial tension, contrasting weak and strong interfacial interactions (left vs right panels) and non-grafted and grafted configurations (top vs bottom panels). In all cases, the tensile response depends on surface geometry, as reflected by the separation of the stress–strain curves. Furthermore, the magnitude of this variation is strongly modulated by interfacial interaction strength and interfacial configurations, as evidenced by the differences across panels.

Under weak interfacial interaction (${\varepsilon _{{\mathrm{np}}}}$ = 0.5 kcal/mol), i.e. Figures 2(a) and (c), variations in surface roughness produce only modest changes in the stress–strain response. Although higher-amplitude rough surfaces exhibit slightly elevated stress over portions of the deformation range, the overall differences among the curves remain limited. This behavior indicates that, in the absence of strong interfacial attraction, roughness alone is insufficient to sustain effective stress transfer and mechanical reinforcement. Interfacial sliding and limited chain constraint reduce the ability of geometric features to contribute to load bearing, resulting in only marginal reinforcement. This observation is consistent with prior studies [16, 18].

In contrast, under strong interfacial interaction (${\varepsilon _{\mathrm{np}}}$ = 2.5 kcal/mol), i.e. Figures 2(b) and (d), the influence of surface roughness becomes much more pronounced. The stress–strain curves show more observable differences over the whole range, with rough surfaces consistently exhibiting higher stress than the smooth reference. This effect is most evident for high-amplitude roughness, where P16H displays the highest stress response among the roughness series. The enhanced response reflects improved interfacial stress transfer: The higher stress response under strong interfacial interaction is consistent with reduced interfacial mobility and more effective load transfer through the rough surface. Because interfacial slip and local stress were not measured directly, this mechanism is inferred from the combined mechanical and MSD results, thereby amplifying the mechanical effect of roughness [16, 18].

The effect of grafting is assessed by comparing non-grafted and grafted systems under identical interfacial interaction (figures 2(a) vs. (c) and (b) vs. (d)). Grafting systematically increases the tensile response for a given surface geometry. This enhancement is observed across both interaction strengths, but is most pronounced when combined with strong interfacial interactions and high-amplitude roughness. Among all the cases considered herein, the grafted P16H system with strong interfacial interaction exhibits the highest stress response. Polymer grafting creates permanent nanoparticle–polymer connectivity and alters interfacial segmental dynamics [17, 18, 22]. The conformations and local organization of grafted chains depend on the grafting architecture and interfacial environment [25, 28, 29]. These structural changes can influence the formation and organization of interchain entanglements and other topological constraints [23, 24]. Consequently, polymer grafting can enhance the mechanical and viscoelastic response of polymer nanocomposites by constraining chain deformation and promoting stress transfer across the nanoparticle–polymer interface [26, 30, 31]. When coupled with a corrugated surface, this connectivity provides additional constraint and mechanical interlocking, resulting in a synergistic increase in tensile resistance. These results demonstrate that surface roughness and interfacial configuration act cooperatively in governing interfacial stress transmission and overall mechanical reinforcement in polymer nanocomposites.

**Figure 3.**
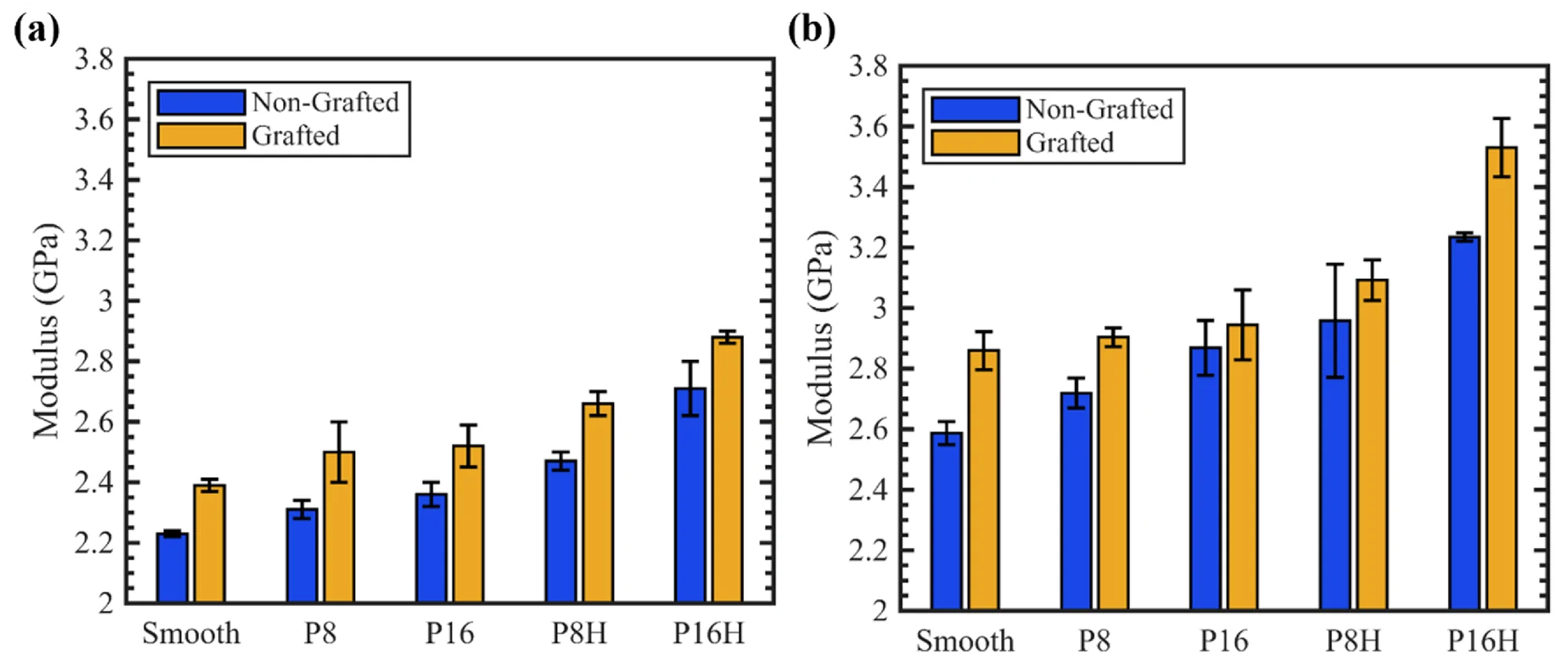
Elastic moduli of nanoparticle-PMMA nanocomposites with different surface geometries under (a) weak and (b) strong interfacial interaction, comparing non-grafted and grafted systems. The modulus is determined independently from the initial linear region of the engineering stress–strain curve for each of three independently prepared and equilibrated simulations. The reported values are the means of the three independently determined values, and the error bars represent their standard deviations.

The elastic moduli of the PMMA nanocomposites are shown in figure 3, with panels (a) and (b) corresponding to weak and strong interfacial interactions, respectively. In both interaction regimes, the modulus tends to be higher for the higher-amplitude rough architectures, although the response also depends on surface periodicity and effective grafting density. This dependence is relatively weak under low interaction strength but becomes more pronounced under strong interfacial interaction. Among all systems, P16H with strong interfacial interaction exhibits the highest modulus, indicating that surface roughness, interfacial adhesion, and interfacial connectivity act cooperatively to maximize stiffness.

The influence of surface geometry observed here is qualitatively consistent with previous findings for rough plate-like fillers, in which surface asperities combined with sufficiently strong interfacial interactions restrict interfacial polymer mobility and enhance stress transfer [16]. Increasing roughness enhances geometric confinement. As a result, local deformation becomes more affine with the nanoparticle surface, increasing resistance to small-strain deformation. We note that plate-like and spherical nanoparticles differ substantially in their overall curvature, which can affect polymer packing, interfacial contact, and chain confinement. In the present study, the overall spherical geometry and equivalent nanoparticle diameter are held approximately constant across all systems, and nanoparticle surface curvature is not independently varied. Comparison with prior plate-like systems is therefore intended only to support the general interfacial mechanism and does not imply quantitative equivalence between nanofiller geometries or that curvature effects are negligible. Separating the respective contributions of nanoparticle curvature and surface nanoasperity would require a systematic study in which these parameters are independently controlled.

Prior studies have also shown that surface geometry and interfacial interaction strength strongly influence chain mobility, confinement, and slip-mediated load transfer in polymer nanocomposites [16, 17, 43]. Under weak interfacial interaction, this geometric constraint leads to only modest stiffening, as chains can partially disengage from the surface during deformation. In contrast, under strong interaction, chains remain more tightly coupled to the surface, allowing roughness features to act as effective anchoring sites that suppress interfacial slip and enhance load transfer.

Grafting provides an additional stiffening mechanism by creating permanent interfacial connectivity within the interphase. In the present simulations, the grafted systems exhibit higher elastic moduli than their non-grafted counterparts for a given surface geometry. This trend is consistent with previous studies showing that polymer grafting can enhance the mechanical and viscoelastic response of polymer nanocomposites [26, 30, 31]. This effect becomes more pronounced with increasing roughness, indicating a cooperative mechanism: surface roughness increases geometric constraint, while grafting stabilizes interfacial coupling. This synergy is most evident in the P16H system, where large surface corrugation combined with grafting produces the greatest enhancement in stiffness.

### 3.2. Interfacial polymer mobility

To quantify polymer mobility near the nanoparticle surface, the mean-squared displacement (MSD) of polymer beads initially located within a common nanoparticle-centered equatorial region is calculated for the non-grafted and grafted systems. More specifically, the sampling region is defined as a 20 Å-thick interfacial shell extending from the nanoparticle surface to an outer radius of 70 Å.

**Figure 4.**
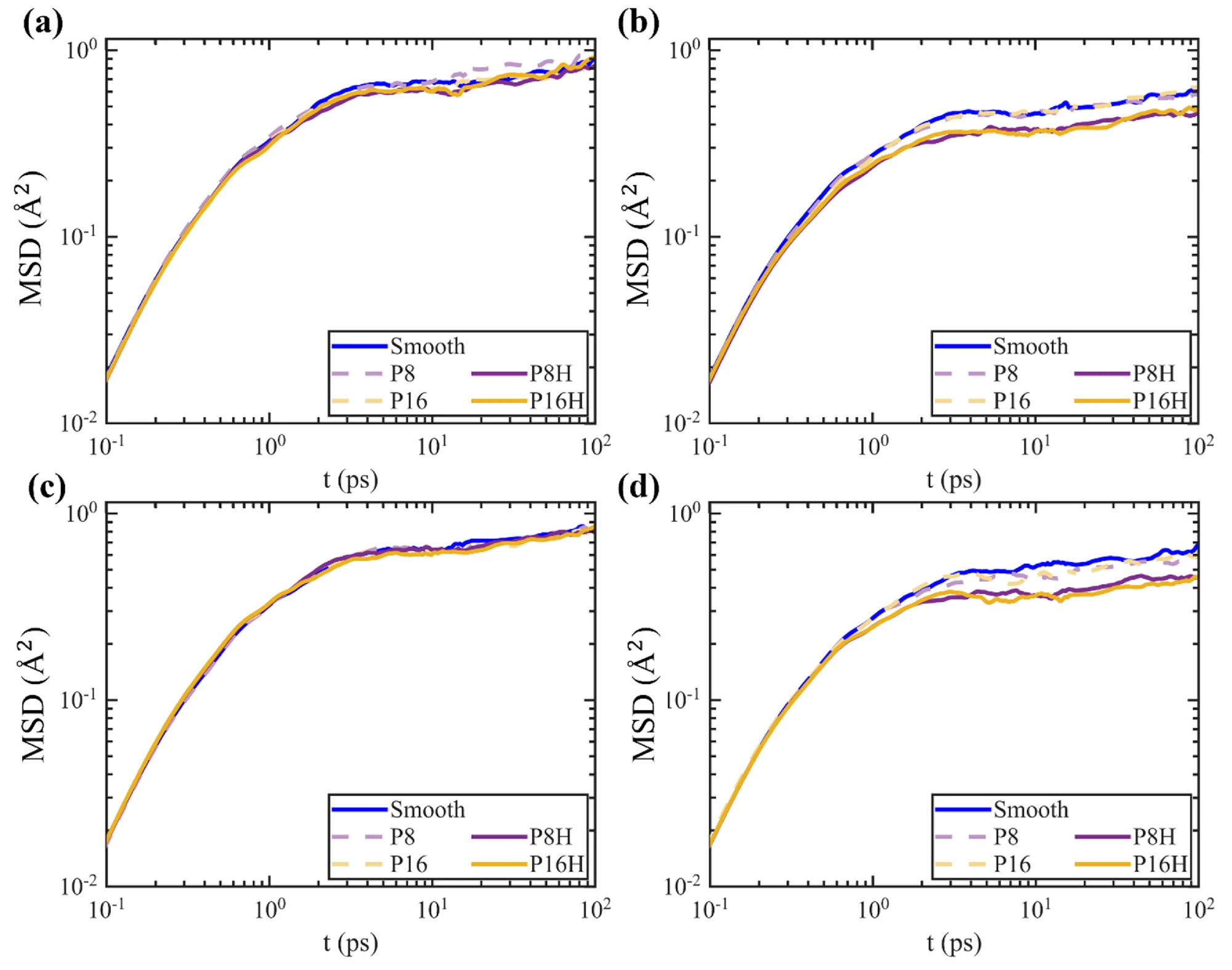
Interfacial MSD analysis of polymer beads initially located within a common nanoparticle-centered equatorial region. Results are shown for (a) non-grafted systems with weak interfacial interaction, (b) non-grafted systems with strong interfacial interaction, (c) grafted systems with weak interfacial interaction, and (d) grafted systems with strong interfacial interaction.

As shown in figures 4(a) and (c) for weak interfacial interaction, the MSD curves are nearly overlapping throughout the investigated time range, indicating little difference in interfacial polymer mobility among the surface geometries. Under the strong interfacial interaction in figures 4(b) and (d), the higher-amplitude P8H and P16H systems exhibit lower intermediate- and long-time MSD values than the smooth reference, whereas the P8 and P16 systems remain comparatively close to the smooth system. These results indicate that strong interactions combined with high-amplitude surface roughness more effectively restrict polymer mobility within the selected interfacial region.

The differences between the non-grafted and grafted MSD curves are comparatively modest (figure 4(a) vs. (c) and (b) vs. (d)). The results indicate that the additional reinforcement associated with grafting is not accompanied by a further reduction in equilibrium interfacial mobility. Therefore, the additional reinforcement associated with grafting cannot be explained solely by changes in equilibrium interfacial mobility. Because grafting introduces permanent nanoparticle–polymer connectivity that may alter the topology of the polymer network, we next use primitive-path analysis to examine how grafting and surface geometry affect the population and geometry of topological constraints.

### 3.3. Primitive-path analysis of topological constraints

**Figure 5.**
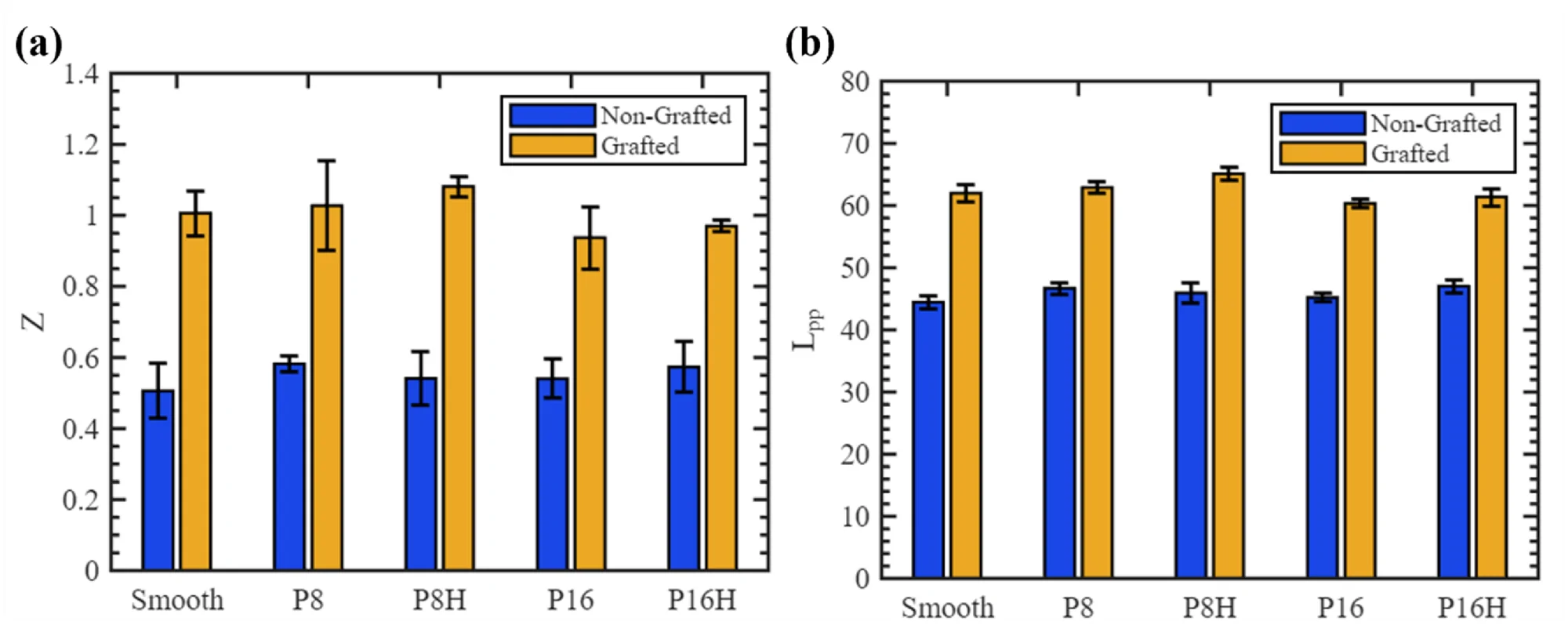
Z1+ primitive-path analysis of various nanocomposites: (a) mean number of kinks per chain, Z, and (b) mean primitive-path contour length, ${L_{\mathrm{pp}}}$, for non-grafted and grafted systems. Each descriptor was calculated separately for three independently prepared and equilibrated configurations. The reported values are the means of the three configuration-level results, and the error bars represent their standard deviations.

To probe the molecular origin of the observed reinforcement, primitive-path analysis is carried out on equilibrated configurations using the Z1+ package [40]. We note that the interfacial MSD analysis above provides complementary spatially selected information on polymer mobility, whereas the primitive-path analysis characterizes changes in the global topology and contractibility of the constrained polymer network. The resulting descriptors, Z and ${L_{\mathrm{pp}}}$, are summarized in figure 5 for the weak interfacial interaction case; similar trends are observed under strong interfacial interaction.

Surface roughness produces a modest increase in topological constraints relative to smooth reference. However, this trend is not strictly monotonic across the roughness series, as shown in figure 5(a). Grafting induces a substantially larger topological shift than surface roughness. For every surface geometry, the grafted system exhibits substantially higher Z than its non-grafted counterpart, demonstrating that tethered chains generate a more strongly constrained polymer network [23, 24, 44]. Mechanically, this increased constraint density suppresses chain pullout and interfacial sliding, thereby enhancing stress transfer between the nanoparticle and matrix and resulting in increased stiffness.

The ${L_{\mathrm{pp}}}$ results in figure 5(b) provide complementary insight into the primitive-path network. Given that ${L_{\mathrm{pp}}}$ depends on both topological constraints and chain conformations (e.g. end-to-end distance and spatial distribution), ${L_{\mathrm{pp}}}$ does not vary monotonically with roughness. In particular, increased constraint can reduce primitive-path contraction during minimization, leading to larger or non-monotonic ${L_{\mathrm{pp}}}$ values. Accordingly, ${L_{\mathrm{pp}}}$ is interpreted here as a descriptor of network geometry and contractibility rather than a direct measure of entanglement density.

Grafted systems also exhibit larger ${L_{\mathrm{pp}}}$, consistent with reduced primitive-path contraction due to graft-induced chain stretching and enhanced interchain constraints. It is worth noting that the grafted series does not show a strictly monotonic dependence of Z and ${L_{\mathrm{pp}}}$ on surface roughness. The mechanically strongest systems do not necessarily coincide with the largest global Z or ${L_{\mathrm{pp}}}$ values. This indicates that tensile reinforcement and stiffness cannot be attributed solely to entanglement density, and that actual reinforcement depends on how effectively constraints are coupled to load-bearing pathways. Nevertheless, the combined stress–strain, modulus, MSD, and primitive-path results support a cooperative interpretation in which grafting increases global topological constraints, while high-amplitude roughness under strong interactions is associated with reduced interfacial chain mobility and more effective load transfer.

### 3.4. Clustered grafting and directional mechanical response

In the cluster-grafted system, the grafting sites are concentrated into two opposing surface domains aligned along the y-direction rather than uniformly distributed over the nanoparticle surface. The cluster-grafted and uniformly grafted smooth systems both contain the same number of polymer chains, all of which are covalently grafted to the nanoparticle. The nanoparticle geometry, polymer chain length, interfacial interaction, graft-bond parameters, and overall composition are identical. Thus, the only designed difference between the two systems is the spatial distribution of grafting sites. Tensile deformation is applied along the x-, y-, and z-directions, shear deformation is applied in the xy, xz, and yz planes to evaluate the directional mechanical response.

**Figure 6.**
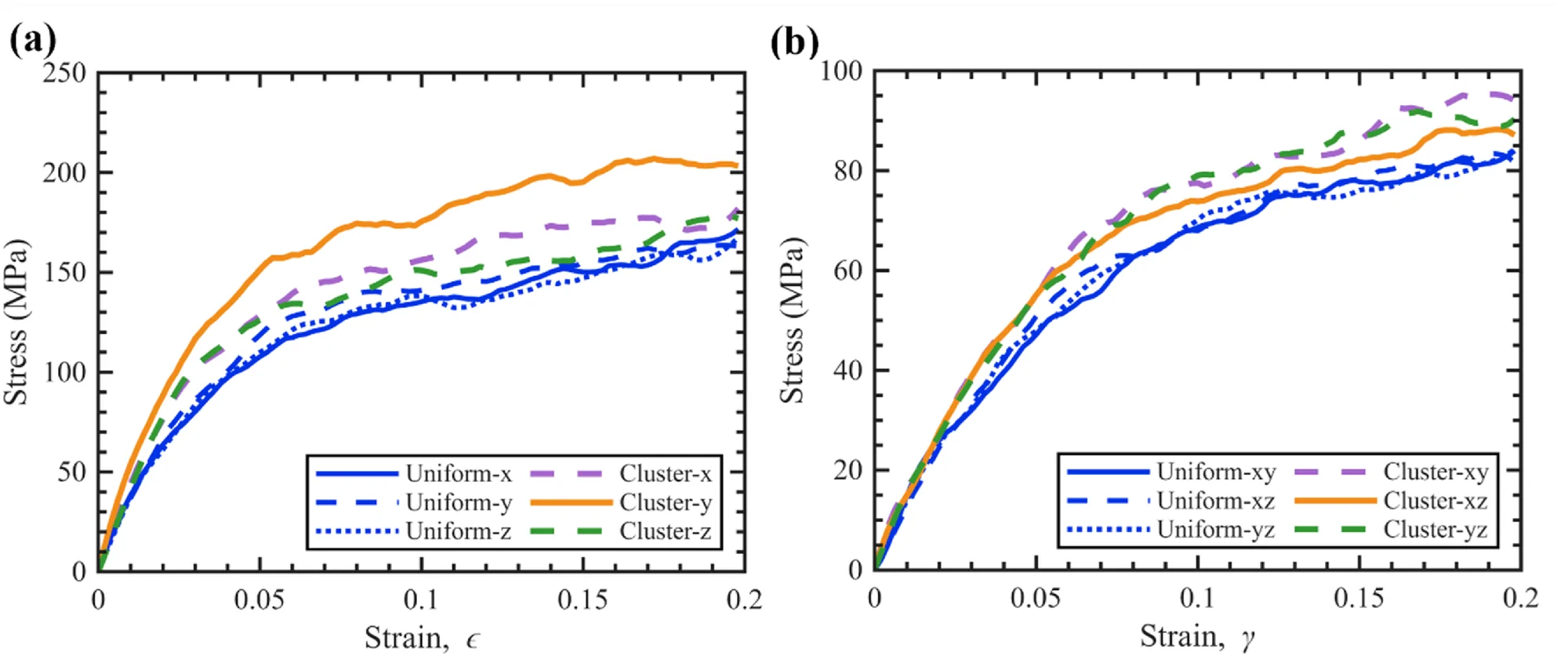
Stress–strain responses of uniformly grafted and cluster-grafted smooth systems under (a) uniaxial tension and (b) shear.

Under uniaxial tension (figure 6(a)), the cluster-grafted system exhibits a clear dependence on loading direction. The highest tensile response is obtained for loading along the y-direction, parallel to the axis connecting the two graft-rich domains. In contrast, loading along the x- and z-directions produces comparable tensile responses, consistent with their nominal equivalence as transverse directions. Small differences between the two transverse curves are attributed to configurational-dependent heterogeneity arising from discrete graft-site placement, finite chain number, polymer packing, and equilibrated chain conformations.

A similar directional dependence is observed under shear deformation, as shown in figure 6(b). Shear deformation applied in planes that engage the clustered regions more directly (e.g. xy and yz plane) produces higher stress levels than xz plane. The differences among the shear planes indicate that the effect of clustered grafting depends on how the imposed deformation engages the graft-rich surface domains.

The directional response is consistent with the heterogeneous interfacial connectivity created by the clustered graft distribution. Whereas uniformly grafted systems have a comparatively homogeneous interphase and a more isotropic load-transfer network, clustered grafting produces graft-rich domains separated by graft-poor regions. This spatial heterogeneity leads to directional variations in interfacial connectivity and polymer constraint. When the loading direction is parallel to the cluster axis, the covalently tethered chains in the graft-rich domains provide persistent connectivity and locally increase resistance to deformation, thereby promoting stress transmission along directions that more strongly engage these domains. By contrast, loading along the nominally equivalent transverse directions engages these domains less directly and produces comparable, weaker responses. Small differences between the two transverse directions are attributed to configurational heterogeneity and are not interpreted as an intrinsic distinction between them.

Primitive-path analysis was also performed for the cluster-grafted system using the same Z1+ procedure applied to the uniformly grafted and non-grafted systems. The cluster-grafted system exhibits Z = 1.87 and ${L_{\mathrm{pp}}}{\text{ }}$ = 86.2 Å, compared with Z = 1.09 and ${L_{\mathrm{pp}}}{\text{ }}$ = 64.0 Å for the uniformly grafted smooth system and Z = 0.51 and ${L_{\mathrm{pp}}}{\text{ }}$ = 45.2 Å for the non-grafted smooth system. The higher Z value indicates a larger number of global topological constraints in the cluster-grafted system, whereas the larger ${L_{\mathrm{pp}}}$ reflects reduced primitive-path contraction and an altered constrained-network geometry. Together with the direction-dependent tensile and shear responses, these results support the conclusion that clustered grafting modifies the constrained polymer network and produces load-direction-dependent responses. Nevertheless, both quantities are global scalar descriptors and do not directly resolve the spatial location or orientation of the constraints.

Because local stress, interfacial slip, and spatial constraint density are not measured directly, we interpret our results from the directional mechanical response and the accompanying global primitive-path analysis rather than demonstrated through a direct local measurement. Nevertheless, this interpretation is consistent with prior studies showing that graft architecture influences interpenetration, entanglement formation, and deformation behavior in polymer nanocomposites [22–31, 44, 45], as well as with observations of anisotropic organization and directional interactions in grafted nanoparticle systems [26, 46, 47].

These results indicate that clustered grafting provides a means of tuning not only the magnitude of reinforcement but also its directional character. Surface roughness and uniformly distributed grafting primarily influence the overall mechanical response, whereas clustered grafting introduces heterogeneous interfacial connectivity and preferential load-bearing directions. Such control may be useful in nanocomposites subjected to multiaxial or nonuniform loading, where graft-rich domains could be strategically oriented to enhance mechanical resistance along preferred loading directions [44–47].

### 3.5. Effect of graft-site placement on interfacial reinforcement

Having established the roles of surface roughness, interfacial interactions, and grafting architecture, we next examine how the spatial placement of grafting sites to specific roughness features affects the mechanical response. To isolate this effect, an auxiliary set of simulations is performed for the P8 surface geometry using two chain lengths, $N = 100{\text{ }}$ and N=50. The shorter-chain system serves as a robustness check to determine whether the effect of graft-site placement persists across different chain lengths and global constraint states. These simulations therefore serve as a comparative sensitivity analysis rather than as a direct extension of the full surface geometry series.

**Figure 7.**
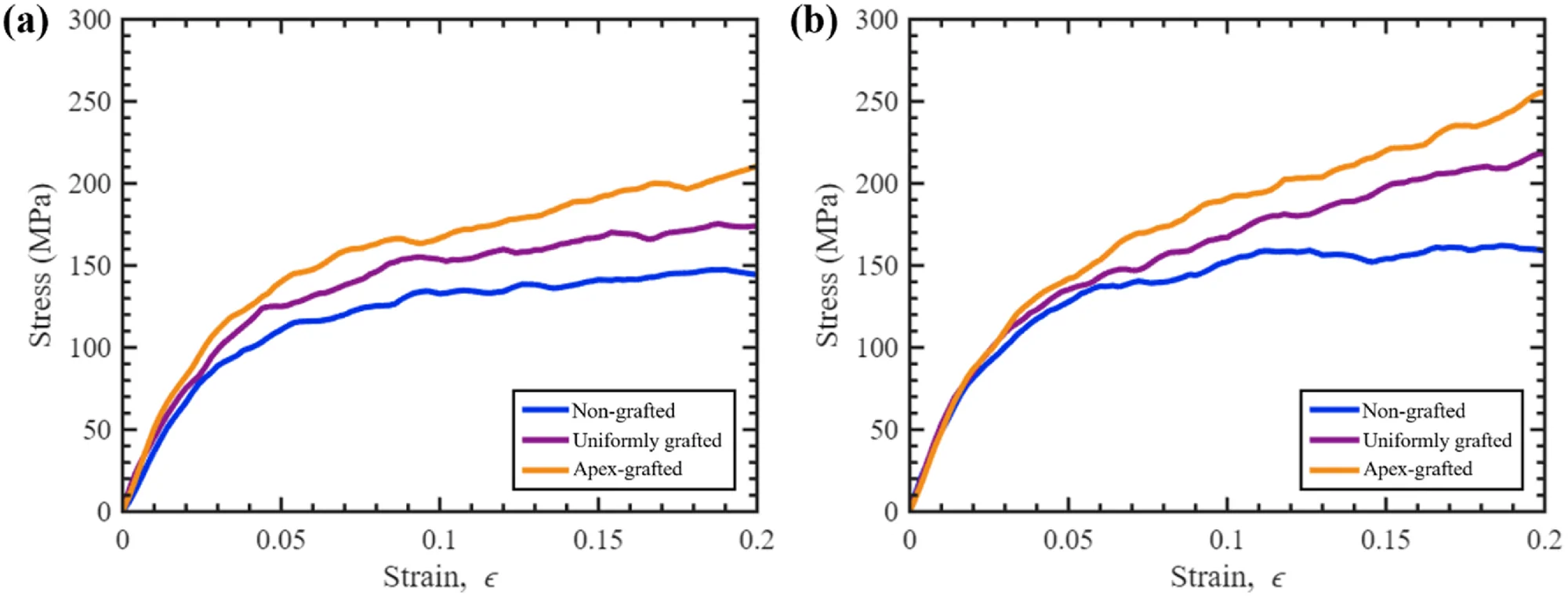
Tensile stress–strain responses of P8 nanocomposites with different grafting architectures at two PMMA chain lengths: (a) N=100 and (b) N=50. The non-grafted, uniformly grafted, and apex-grafted systems are compared.

Figure 7 compares the tensile stress–strain responses of three interfacial configurations: the non-grafted P8 system, the uniformly grafted P8 system, and the apex-grafted P8 system. For both chain lengths, the apex-grafted system exhibits the highest tensile response over most of the deformation range, followed by the uniformly grafted system, while the non-grafted system shows the lowest response. The differences are relatively small at low strain but become more apparent at intermediate and large strain. The same ordering across both chain lengths indicates that the effect of apex-targeted grafting is not restricted to a single polymer chain length. These results show that grafting increases the tensile response relative to the non-grafted P8 system and that positioning grafting sites at the surface apexes produces an additional increase relative to an approximately uniform graft-site distribution.

The higher tensile response of the apex-grafted system indicates that the spatial relationship between grafting sites and nanoparticle surface geometry influences reinforcement. The convex apexes are geometrically prominent surface features and placing covalent grafting at these locations may couple the tethered chains more effectively to the rough interface during deformation. The observed response indicates apex-grafted architecture more effectively engages the roughness features under load bearing. This result is consistent with prior studies showing that roughness features can act as mechanical anchoring sites when interfacial coupling is maintained [16, 43], and that grafting architecture and spatial heterogeneity influence interfacial stress transfer and deformation behavior [19–22, 28, 48].

It is important to emphasize that the effect of grafting site distribution remains secondary to the effects of interfacial interaction strength, nanoparticle surface geometry, and the introduction of covalent grafting identified in the preceding sections. Overall, these results show that graft-site distribution is an additional grafting feature that can be coordinated with nanoparticle surface geometry to tune mechanical reinforcement.

## 4. Conclusions

In this study, CG MD simulations are used to investigate how nanoparticle surface roughness, interfacial interaction, and grafting architecture jointly affect the mechanical response of nanoparticle-PMMA nanocomposites. The results show that systematic changes in surface geometry produce clear and measurable differences in mechanical response, but its effect depends strongly on the interfacial interaction strength. Under weak interfacial interaction, the differences among the smooth and rough surfaces are modest. Under strong interactions, nanoparticles with a higher-amplitude rough surface exhibit higher tensile stresses and elastic moduli and slower interfacial MSDs than smooth case. These trends are consistent with more restricted interfacial polymer mobility and more effective load transfer through the rough interface. Among the systems examined, the grafted P16H system under strong interfacial interaction exhibits the highest elastic modulus.

Introducing grafting enhances reinforcement for each surface geometry and produces a larger change in the global primitive-path network than variations in surface geometry, whereas its effect on interfacial MSD is comparatively modest. Primitive-path analysis shows that grafted systems exhibits higher mean numbers of kinks per chain and longer primitive-path contour lengths, indicating a greater population of global topological constraints and reduced primitive-path contraction. However, the mechanically strongest systems do not necessarily exhibit the largest primitive-path descriptors. Therefore, reinforcement cannot be attributed to the global constraint population alone; it also depends on how effectively those constraints are coupled to interfacial load-bearing pathways.

The spatial distribution of grafting sites provides an additional means of controlling mechanical response. Relative to the uniformly grafted smooth reference, clustered grafting produces direction-dependent tensile and shear responses, with the highest tensile response occurring along the cluster axis. For the P8 nanoparticle, the apex-grafted case produces a higher tensile response than the uniformly grafted and the non-grafted baseline at two levels of chain lengths examined. These results show that aligning graft-rich regions with the loading direction or with mechanically active surface features can modify the magnitude and directional character of reinforcement.

Overall, this study identifies surface roughness, interfacial interaction, and grafting architecture as cooperative design variables for controlling mechanical reinforcement in polymer nanocomposites. More broadly, this work provides a molecular-level framework for designing engineered nanofillers, in which surface geometry and grafting architecture are simultaneously tailored to optimize mechanical performance.

## Data Availability

The data that support the findings of this study are available upon reasonable request from the authors.
